# Characterization of sago tree parts from Sentani, Papua, Indonesia for biomass energy utilization

**DOI:** 10.1016/j.heliyon.2024.e23993

**Published:** 2024-01-05

**Authors:** Benny Susanto, Yohanis Tangke Tosuli, Adnan, Cahyadi, Hossein Nami, Adi Surjosatyo, Daffa Alandro, Alvin Dio Nugroho, Muhammad Ibnu Rashyid, Muhammad Akhsin Muflikhun

**Affiliations:** a Department of Mechanical Engineering, Universitas Indonesia, Kampus UI Depok, 16424 Indonesia; b PT. PLN (Persero) Research Institute, Jakarta, Indonesia; c BRIN, Serpong, Indonesia; d SDU Life Cycle Engineering, Department of Green Technology, University of Southern Denmark, Campusvej 55, Odense M, 5230, Denmark; e Mechanical and Industrial Engineering Department, Gadjah Mada University, Indonesia; f Center for Advanced Manufacturing and Structural Engineering (CAMSE), Gadjah Mada University, Indonesia; g Center of Energy Studies (PSE), Gadjah Mada University, Indonesia

**Keywords:** Sago waste, Kinetic sago bark parameters, Biomass, SEM–EDX, TGA–DTA

## Abstract

Biomass derived from organic waste in industrial processes is an effective method to mitigate the negative impacts of agricultural waste materials. In Sentani, Papua, one such potential biomass source is sago tree waste. This study characterized the waste from the bark, middle, and inner parts of the sago tree to evaluate its biomass energy potential. Scanning electron microscopy with energy-dispersive X-ray (SEM-EDX) analysis of the complete sample revealed that oxygen, carbon, and silicon were the primary elements, with carbon content ranging from 30.75 % to 38.87 %. This indicates that all parts of the sago plant have the potential to be used as biomass fuel. Thermogravimetric analysis (TGA) results showed that the inner section of the sago had the lowest moisture content at approximately 13.3 %, followed by the outer part at 42 % and the bark at 55 %. The inner section had the highest lignin content, approximately 37 %, and exhibited the slowest thermal degradation in the differential thermal analysis (DTA) profile. The outer and bark parts of the sago were more reactive in stage II of the DTA profile, suggesting a higher concentration of cellulose and hemicellulose compared to lignin, making them suitable for gasification and pyrolysis. The heating value of sago bark was determined to be 12.85 MJ/kg (adb). These findings underscore the potential of sago waste as a renewable energy source, particularly in remote areas.

## 1 Introduction

The sago palm (Metroxylon sagu) is a sustainable agricultural plant in tropical areas. It has been identified as a highly beneficial solution for societal needs, particularly in the realms of agricultural and plant-based products. This recognition stems from its economic viability, contribution to stable agroforestry systems, and environmental sustainability [1,2]. Sago commonly grows in wetland areas where other crops cannot thrive, often due to poor soil and drainage conditions [3,4]. In Indonesia, particularly in Papua, sago and its products play a crucial role in supporting food security. The Estate Crop Statistics of Indonesia from 2015 to 2017 provide insights into smallholder, public, and private estate areas, along with production and productivity data at district and provincial levels [[5], [6], [7], [8]]. This includes information on volume, import and export values, regional and global market prices, and world balance data for various goods. Sago is a significant food product in Indonesia [[9], [10], [11]]. The World Health Organization recognizes Indonesia as one of the world leaders in terms of the number of sago plantations. Indonesia has the largest sago-producing area globally, covering approximately 1.128 million hectares, which is 51.3 % of the world's total of 2.201 million hectares of sago. This represents more than half of the global sago production [8]. Data from the Indonesian Plantation Statistics, documented in the Indonesian Sago Plantation Book, corroborate this fact. As of 2017, the sago plantation area in Indonesia had expanded to 219,978 ha, producing a total of 487,643 tons. These plantations are distributed across various regions, including Sumatra (106,179 ha), Kalimantan (9181 ha), Maluku and Papua (87,264 ha), and Sulawesi (17,354 ha) [12].

Focusing on South Sulawesi, the sago plantation area spans 3896 ha, yielding 2560 tons with a productivity rate of 1217 kg per hectare annually. This region demonstrates significant potential for sago cultivation as an alternative food crop, potentially replacing rice. A single sago plant can yield approximately 200–250 kg of wet sago each year, which equates to 25 tons of dry starch per hectare. This starch contains 84.7 g of carbohydrates per 100 g [8,13]. Sago starch extraction employs various methods, ranging from traditional to semi-modern and modern techniques. While the fundamental principles of these methods are similar, they differ in operation scale and technological aspects [14]. The extraction process includes felling, splitting, and skinning mature sago palms, grating the sago stalks to extract starch, followed by drying and packaging. Additionally, the consistent supply and abundance of waste materials from the agro-industrial process present exploitable potential due to ongoing production [15]. The extraction of sago starch necessitates substantial water use, resulting in wastewater and solid waste production. In traditional manufacturing, this solid waste comprises sago pulp (solid sago waste) and bark, which can be processed to obtain sago fibers [16]. Various components of the sago plant, such as leaves, seeds, fruits, and stems, are used to harvest these fibers. Water is sprayed through perforated pipes onto the reel's body, flushing out the rasped pith and starch grains [17].

Waste fibers are separated and discharged from the lower end of the washing cylinder, while starch-laden water passes through a coarse wire screen, removing most of the fiber before sedimentation in cement tanks or wooden troughs [[17], [18], [19], [20], [21], [22], [23]]. The renewable and biodegradable nature of these materials offers significant advantages. All-natural fibers are biobased and decompose naturally in the environment at the end of their life cycle [[24], [25], [26], [27], [28], [29], [30]]. The renewability and biodegradability of these materials provide significant benefits. Natural fibers, derived from organic sources, can naturally break down in the environment at the end of their lifecycle [31].

Natural fibers, including those derived from sago, constitute a composite material. This material comprises a soft, amorphous matrix composed of lignin and hemicellulose, along with stiff, crystalline cellulose microfibrils [[32], [33], [34], [35], [36], [37]]. The bark of the sago plant, primarily containing lignin, holds potential as a substitute for wood. However, the use of sago bark as an alternative raw material for wood remains relatively limited. Therefore, researchers are investigating its physical properties, such as moisture content, acid and base resistance, compressive strength, and modulus of rupture. These properties are crucial in determining its suitability for various applications and could lead to its utilization as a wood substitute. Sago bark waste, the outer part of the sago stem, is predominantly composed of sclereid cells. For every ton of sago flour produced, approximately 0.75 tons of sago bark is generated as solid waste [38]. Data from the Department of Statistics Malaysia indicated that in 2011, the sago-producing country produced around 52,000 tons of sago flour, a figure expected to rise with increased production. This increase could exacerbate pollution, leading to marine biota disruption and water pollution-related poisoning if current sago waste processing methods persist [39].

Sago bark waste is the outer part of the stricter sago stem or the peripheral part dominated by sclereid cells [40]. Every ton of sago flour produced generates approximately 0.75 t of sago bark as a solid waste. Therefore, sago bark is a potential source of natural fiber [41]. Sago starch is a high-yield starch with significant development potential [42,43]. Starch, as one of the most studied eco-friendly, affordable, biodegradable, and thermoplastic natural polymers, shows great promise [44]. In various regions of Indonesia, sago starch yield varies significantly, ranging from 150 to 700 kg per trunk, with an average yield of 300 kg of fresh starch [45]. Consequently, sago bark presents a viable source of natural fiber. Major sago palm growers include Malaysia, Indonesia, and Papua New Guinea [46].

While there have been studies analyzing sago characteristics and evaluations, research specifically focused on sago from Papua, Indonesia, remains limited. Consequently, data on sago plants cultivated in Papua is scarce. This study aims to comprehensively analyze the characteristics of the sago tree, with Papua, Indonesia, as the primary research site. Sago samples were collected from four districts in Sentani: Bata, Yakari, Dondo, and Yebha. The analysis utilized scanning electron microscopy (SEM) to examine morphological differences and energy-dispersive X-ray (EDX) analysis to determine the elemental composition of the sago waste. Thermogravimetric analysis (TGA) and differential thermal analysis (DTA) were also conducted to estimate the cellulose, hemicellulose, and lignin content in the inner, outer, and bark portions of the sago. The results of this study are valuable for further research related to the pyrolysis or gasification processes in electricity generation using biomass products. Moreover, this work provides data on the distinctive characteristics of the sago plant from Papua, an area where data has been limited until now.

## 2 Materials and methods

### 2.1 Research site

Sago waste agriculture product site was located near Sentani Lake in Jayapura City, Papua Province. This location was selected based on previous research by Yamamoto et al. and others [[47], [48], [49], [50]]. The research, conducted from 2003 to 2007 and 2011 to 2012, spanned several districts, including Ifar Besar, Kleublouw, Yahim, Hawai, Yabaso, and Ariau [[47], [48], [49], [50]]. In this area, the process of gasifying sago waste for syngas production is not yet established. Surveys of this location were conducted from November 2003 to 2020 by Yamamoto, and we continue to explore and characterize sago waste from sago pith industry [48,50].

### 2.2 Varieties of sago palms

From 2021 to 2022, the initial research on the sago variety was conducted in collaboration with the Bureau of Agricultural Technology Assessment Papua in Sentani. The varieties of sago used in this research were based on the locations in six districts. Out of five identified varieties of sago palm growing in these conditions, only four were selected for study due to material availability. In Sentani, there were three intra-species spiny sago palm varieties (Yakari, Bata, and Dond) and two unspiny varieties (Yebha and Ojokuru). Yebha was the dominant type. The clusters were distributed sporadically with a coefficient of variance of 24.3.

### 2.3 Materials

**Fig. 1:**
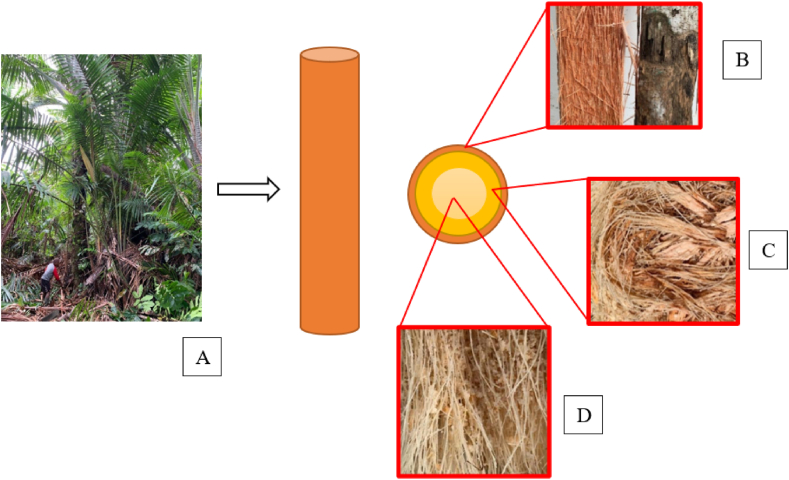
Parts of the sago tree used: (a) tree trunk, (b) bark, (c) middle fiber, and (d) inner fiber.

**Fig. 2:**
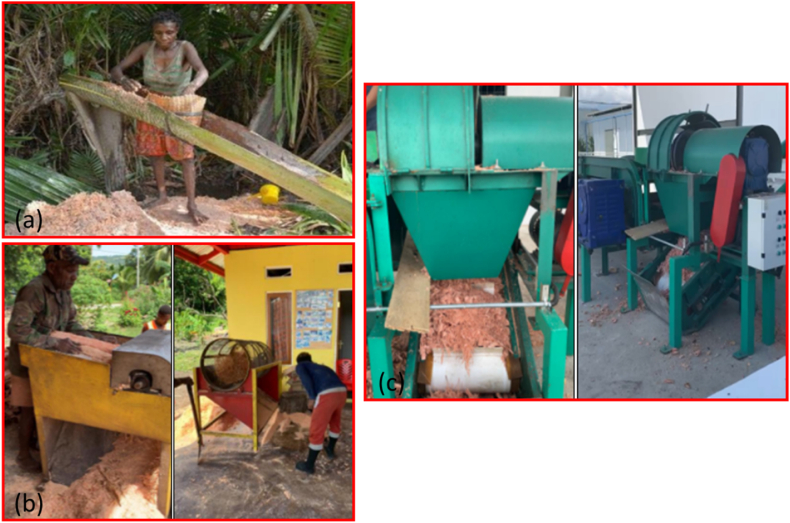
Sago tree processing: (a) traditional process, (b) semi-modern process, and (c) modern process.

Sago sampling specimens were collected from the trunks of the trees. Each trunk was cut at the node of the lowest surviving leaf, and its length was measured from the cut ground point up to the first leaves of the sago plant. The materials researched from the sago trunk included the inner fiber, middle fiber, and bark. The waste from the sago palm used in this research is depicted in Fig. 1(a–d). The part of the fiber utilized is from the previously processed sago tree, specifically the outer, middle, and inner sections of the trunk. Table 1 shows the chart of sago pitch production. In the Sentani Lake area, the sago production process is categorized into three models: traditional, semi-modern, and modern processing. The main differences in these processes in the Sentani Lake area are illustrated in Fig. 2(a–c). In traditional processing, the sago palm is cut into several trunk sections, each approximately 60–100 cm in length. The bark is then removed, and starch extraction is performed near a river or water source. This traditional method results in sago bark, fibers, and pulp as waste, while the sago pith is processed for the food market. Sample designation is based on the district name, followed by the tree part. For instance, samples from the Bata district are designated as "B" (from the first letter), Yakari as "I" (from the last letter), Dondo as "D" (from the first letter), and Yebha as "A" (from the last letter). The tree parts are abbreviated as follows: bark (B), middle fiber (MF), and inner fiber (IF).

**Table 1:** Sample designation according to sago location.

Part of Sago	Part of Sago		
	Bark	Middle Fiber	Inner Fiber
Bata	BB	BMF	BIF
Yakari	IB	IMF	IIF
Dondo	DB	DMF	DIF
Yebha	AB	AMF	AIF

### 2.4 Sago processing

The traditional sago processing method is employed by individual farmers in the districts surrounding Sentani Lake, as outlined in Table 2. The process involves farmers cutting the sago palm in the forest or fields, removing the bark from the trunk, and then processing the sago starch and pulp at a single location. Typically, the solid waste from sago processing is discarded near rivers. These waste materials are not utilized for daily consumption by the farmers. In semi-modern sago processing at Sentani Lake, processing occurs at a central sago community near the districts surrounding Sentani Lake Area. Here, the sago palm is cut from forests or fields, and the bark is removed in several parts. The trunk is ground or rasped using homemade rotary equipment. Then, the fibers are cut, screened with a cylindrical cyclone separation screen, and ground. The resulting material is processed in water to form sago pulp, which is then pressed and filtered to separate the sago starch.

**Table 2:** Sago processing and its results from traditional, semi-modern, and modern processes [53].

No.	Step	Traditional	Semi-modern	Modern	Remark
1	Sago palm logs	✓	✓	✓	
2	Bark removal	✓	✓	✓	Sago bark
3	Dry rasping of pith	✓	✕	✕	
4	Wet maceration of course chip	✓	✕	✕	
5	Sleeving	✓	✓	✕	Fibers
6	Setting and washing of starch slurry	✓	✓	✓	
7	Further purification	✓	✓	✓	
8	Drying	✓	✓	✓	
9	Sago starch	✓	✓	✓	Pulp

**Fig. 3:**
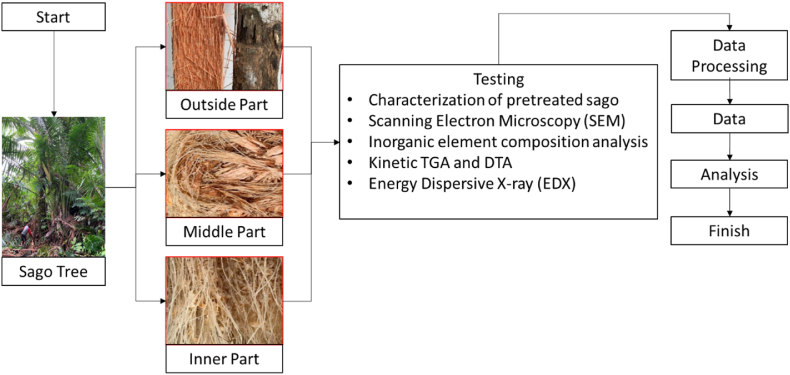
Manufacturing process of specimens and testing.

Modern sago processing starts with cutting the sago palm in a forest or land and cutting the trunk into sections of 60–100 cm each. The process of bark removal up to sago starch production is automated. This method produces only two types of waste: sago bark and pulp. Sago bark is generated during the industrial processing of sago pits. Often, this waste is identified as biomass at least two weeks after processing. Special treatment is required for these samples during packaging and transport to the laboratories. For transportation from Sentani Lake to Jakarta, all samples (four varieties, each with three samples, totaling 12 samples) were packaged in sealed plastic clamps. Fig. 3 provides a schematic of the characterization process.

### 2.5 SEM and EDX analysis

Pretreated sago solid waste samples were analyzed using field emission SEM (FEI Inspect 150) at Universitas Indonesia. Areas observed included the bark, middle fiber, and inner fiber of the sago tree. SEM analyzed the morphology of each part, with the understanding that different morphologies might contain varying percentages of elements. The electrons that backscatter upon interacting with the sago generate characteristic X-rays from each compound, which are then captured by the EDX detector. EDX was employed to determine the inorganic composition of the sago waste in each sample and confirm the presence of C and O elements. EDX analysis has been proven effective for analyzing materials with organic and inorganic elements across nano, meso, micro, and macro sizes [51,52].

### 2.6 Kinetic TGA and DTA

To study the thermal behavior during the pyrolysis of sago biomass, TGA and DTA were employed. The Shimadzu TG–DTA 60H machine was used for this purpose. The sample weights for the inner, outer, and bark portions were approximately 6.5 mg, 11 mg, and 14.5 mg, respectively. The heating temperature ranged from ambient to 800 °C at a rate of 25 °C/min. After reaching 100 °C, the temperature was maintained at this level for 5 min, after which the heating was continued at 25 °C/min.

### 2.7 Proximate and ultimate analysis

Proximate and ultimate analyses were conducted on the sago material samples, with a focus on obtaining the optimal results from the sago bark part.

## 3 Results and discussion

### 3.1 SEM and EDX analyses

**Fig. 4:**
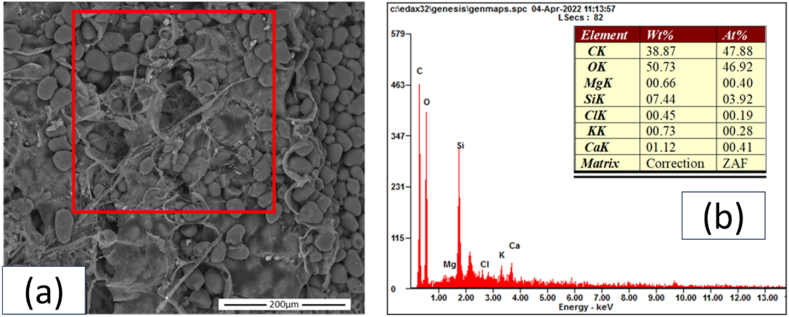
SEM–EDX results from representative samples: (a) SEM image of BB and (b) EDX results from BB.

The SEM results revealed differences in the morphology of sago waste from various districts and locations. EDX analysis was conducted to determine the elemental characterization of the sago waste. Representative SEM images and EDX are shown in Fig. 4(a–b), while a complete set of SEM images is presented in fig. A1. These images demonstrate varying surface morphologies across different types and locations of sago. For instance, the BB sample revealed clearly visible sago granules and starch. Some granules were partially covered by an amyloplast microlayer. In the BMF sample, as shown in fig. A1, a higher concentration of Ca elements was observed compared to samples from the other places, indicated by the presence of white granules on the surfaces. The BIF sample showed similarities, with granules covered by amyloplast in several areas (with the presence of a microlayer). The IB sample exhibited a dense coverage of amyloplast over most of the surface. The IMF sample exhibited a high presence of granules but with no amyloplast on the surface. In the IIF samples, granules were gathered within an amyloplast bag, with clear partitions visible. The DB sample presented amyloplasts with a spider web-like transformation, similar to the DMF sample, where the web covered more than 50 % of the surface, enveloping the granules. The granules were distinctly visible on the surface in the DIF sample, with amyloplast bags noted again in the AB and AMF samples. The AIF samples showed a dense coverage of amyloplast webs on their surfaces.

**Fig. 5:**
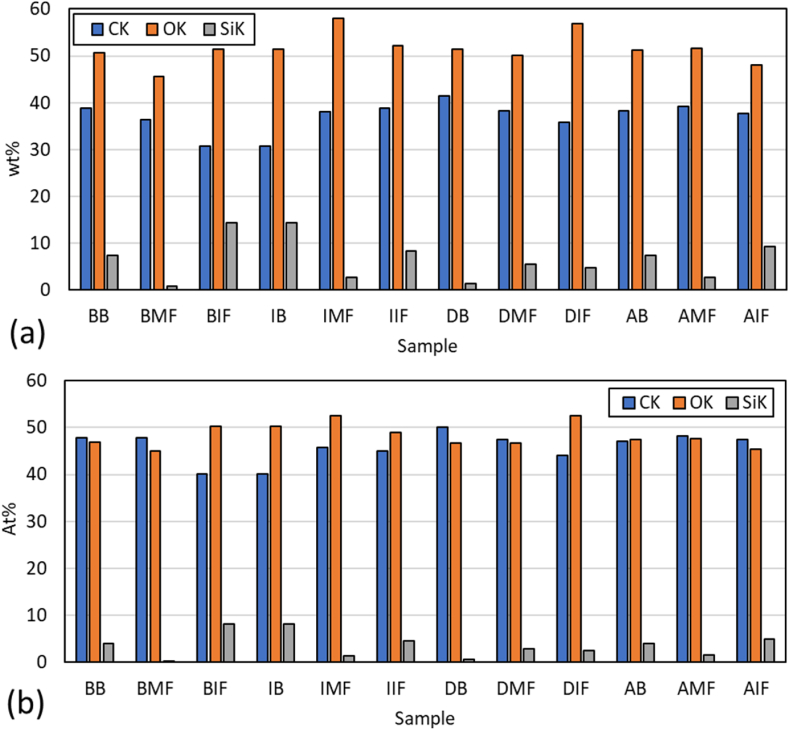
EDX results of sago waste: (a) weight % and (b) atomic %.

EDX results for all samples are shown in Fig. 5(a–b). EDX characterization indicated that the most prevalent elements in the samples were C, O, and Si, with Ca also appearing in high concentrations in some specimens. Other trace elements such as Mg, Al, Cl, and K were also detected. The sago samples revealed varying percentages of elements based on location, as per the EDX results.

In the Bata district, the bark parts exhibited sago particles with dimensions of approximately 30 μm, with some particles forming groups. Natural tissue was observed wrapping around certain areas. The dominant elements in the bark were C, O, and Si, with values of 38.87 wt%, 50.73 wt%, and 7.44 wt%, respectively. The middle fiber parts exhibited smaller particles, approximately 10 μm, with varied colors. These particles were embedded within the middle fiber parts, where the Ca element was present. The predominant elements were C, O, and Ca, with values of 36.45 wt%, 45.67 wt%, and 10.18 wt%, respectively. The inner fiber area featured larger particles, approximately 30 μm in dimension, similar to those in the bark parts. The concentrations of C and O were lower than in the bark and middle fiber parts, but that of Si was higher. The values for C, O, and Si were 30.75 wt%, 51.49 wt%, and 14.45 wt%.

The bark parts of the Yakari sago exhibited particle dimensions of approximately 50 μm, grouped within many tissue formations. In these parts, the C and O contents were the lowest among all the parts, with the presence of Si. The content values for C, O, and Si were 30.75 wt%, 51.5 wt%, and 14.45 wt%, respectively. The middle fiber parts exhibited fewer tissue formations, with particle dimensions of approximately 50 μm. The C and O contents were higher than in the bark part, with Si also present. The content values for C, O, and Si were 38 wt%, 58.1 wt%, and 2.8 wt%, respectively. The inner fiber exhibited more grouped particles but in smaller quantities, with dimensions of approximately 30 μm. The content values for C, O, and Si were 35.9 wt%, 52.2 wt%, and 8.3 wt%, respectively. This part had higher C and O content in the Yakari sago.

Sago from the Dondo district exhibited a distinct morphology compared to other districts. The presence of both tissue and abundant stringing was notable. In the bark parts, large areas of sago string were observed, with particle dimensions of approximately 30 μm. Some areas contained fewer particles than others. The elemental contents of C and O were 41.5 wt% and 51.43 wt%, respectively. In the middle fiber parts, both tissue and stringing of sago were present, with most particles covered by tissues and strings. The majority of particles, approximately 50 μm in dimension, were grouped around the tissue. The values for C, O, and Si were 38.3 wt%, 50.2 wt%, and 5.5 wt%, respectively. In the inner fiber parts, stringing was absent, and there was minimal tissue presence, with a particle size of approximately 40 μm.

Yebha sago exhibited characteristics similar to that of the Bata and Yakari districts, except in the inner fiber. In the bark parts, particles grouped around the tissue, with dimensions of approximately 50 μm. The values for C and O were the highest in these parts, at 38.2 wt% and 51.3 wt%, respectively. Si was also present, with a value of 7.4 wt%. The middle fiber area showed tissue presence, with some particles embedded inside. These particles were approximately 50 μm in dimension, with C and O values at 39.2 wt% and 51.64 wt%, respectively. In the inner fiber area, significant stringing and fibers were observed. Particles in this district were less prevalent than in other parts, with an approximate dimension of 50 μm. The elemental contents of C, O, and Si were 37.66 wt%, 48.1 wt%, and 9.3 wt%.

SEM–EDX analysis proved valuable in evaluating the morphological conditions of the specimens [54]. Most sago parts from different districts showed high C and O content, particularly in the middle fiber parts, likely due to less tissue area and higher particle content. Several samples with tissue presence also indicated Si in the EDX results. The IB, IIF, AB, and AIF samples specifically demonstrated this based on SEM–EDX analysis. The AIF samples exhibited the highest Si content.

### 3.2 TGA and DTA analyses

**Fig. 6:**
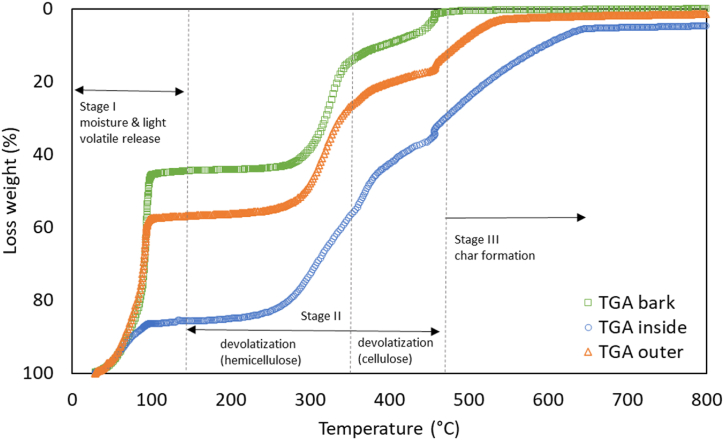
TGA results of bark, inner, and outer sides of sago.

The DTA results (Fig. 6) at point A, using a specimen from the inner part of the sago tree fiber, showed a temperature of 181.34 °C and an output of 16.31 μV. This observation from the graph indicates an endothermic process occurring at the 12th minute, where the specimen absorbs heat. In the TG–DTA tests, approximately 14 mg of the sample was heated from room temperature to 800 °C at a rate of 20 °C/min in an air environment, with a gas flow rate maintained at 50 ml/min. Nitrogen gas was introduced to the analyzer at 105 °C and held for 5 min to remove moisture from the sample. Subsequently, the analyzer was filled with air until a temperature of 800 °C was reached. At a temperature increase to 358.83 °C, an exothermic process occurred, releasing heat from the system, indicated by a graph value of 32.46 μV. Corresponding to this, a decrease was observed in the TGA curve at point A, with a mass loss of 0.73 mg between the temperatures of 57.92 °C and 217.97 °C. At the 26th minute of DTA testing, a temperature increase to 409.69 °C was recorded with an output of 43.19 μV, indicating a shift from heat absorption to heat release. TGA testing at this point showed a mass decrease at 350.09 °C, with the specimen's total mass reducing from 5.55 mg to 3.65 mg. Furthermore, at 44 min, the final DTA output was −18.68 μV, reflecting an inability of the specimen to retain heat due to the temperature increase and the heat release process exceeding its capacity. At this time, TGA results showed the specimen's mass decreased to 0.53 mg. As the temperature increased, the mass loss of the specimen also increased.

The TGA test utilized three types of specimens: bark, inner, and outer parts of the sago tree. The first step involved removing moisture and light volatiles at a temperature of 100 °C. The inner specimen experienced only –an approximately 14 % reduction in total weight, a significant difference compared to the bark and outer specimens. This difference is attributed to the higher moisture content in the inner specimen, resulting in a relatively smaller weight reduction at this temperature compared to the bark and outer specimens, which contained less moisture. During the second and third steps, at temperatures of 200–400 °C, organic cells in the plants began to release volatile compounds, and drying increased, leading to a decrease in specimen weight as the temperature rose. The final heating step, at 500–800 °C, involved the formation of carbon bonds. The bark specimen showed no residual weight at the end of heating; the outer specimen exhibited a weight reduction of 98 %, while the inner specimen, with higher moisture, showed the greatest weight retention with a reduction of 94 %. The energy required to exhaust the moisture was greatest in the specimen with the highest moisture level.

Pyrolysis of biomass involves the variable response of components such as cellulose, hemicellulose, lignin, inorganics, and certain extractives [[55], [56], [57]]. Three distinct phases of sago biomass decomposition are depicted in Fig. 6: moisture and low volatile temperature release (stage I), devolatilization (stage II), and lignin degradation (stage III). Stage I degradation begins between room temperature and 160 °C, corresponding to the elimination of both bound and unbound moisture and lower molecular weight compounds from the biomass. Stage II (160–480 °C) corresponds to the devolatilization of major biomass constituents, namely hemicellulose, cellulose, and some lignin. This phase produces the maximal amount of volatile substances, including hydrocarbons, carbon dioxide, and incombustible gases such as nitrogen and carbon monoxide, and is also known as the active pyrolysis zone. The final stage, at temperatures above 480 °C, corresponds to the degradation of lignin and char formation.

In the first stage, sago bark experienced a substantial weight loss, followed by the outer and inner surfaces. TGA results indicate the moisture content of each sample based on the weight loss at 105 °C. The bark's moisture content was approximately 54.6 %, while the outer and inner parts of the sago contained 42.6 % and 13.3 %, respectively. In the initial phase, between 100 °C and 200 °C, mass loss occurred due to the vaporization of water and the decomposition of low-temperature volatile compounds.

**Fig. 7:**
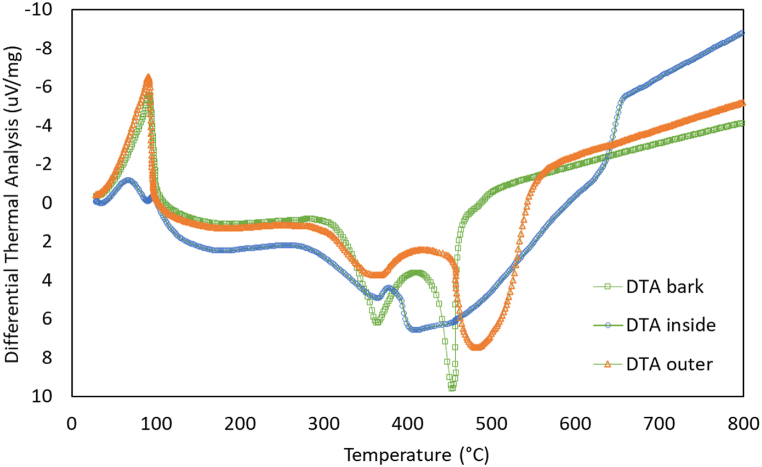
DTA results of bark, inner and outer sides of sago.

Stage II analysis revealed that the inner portion of the sago has approximately 50 % higher hemicellulose and cellulose content compared to its outer and bark parts, which contain approximately 35 %. In stage III, the inner sago was found to have the highest lignin concentration, approximately 37 %, compared to 10 % in the bark and 20 % in the outer sago. Fig. 7 depicts the exothermic (heat release) and endothermic (heat absorption) characteristics of biomass degradation, as determined by DTA curves. The exothermic effect in various biomasses varies due to factors such as chemical structure, component concentration, heating rate, and the presence of hemicellulose, cellulose, and lignin [55,[58], [59], [60]].

In the DTA test, the specimen is heated to 100 °C, entering the endothermic phase, absorbing heat or energy. The outer specimen absorbed the most energy compared to the other two. The bark and outer specimens exhibited more dominant heat absorption than the inner specimens at this temperature. As the temperature increased to 200–400 °C, an exothermic reaction occurred at around 380 °C, where all three specimens emitted heat or released heat energy. The specimen with the highest value was the bark. The bark specimen reached its peak heat release at 450 °C with 10 μV/mg. This temperature was where the bark specimen produced the highest heat energy. The ability of all three specimens to absorb heat energy decreased as the temperature increased during the heating process. Specimens with lower humidity produced higher heat energy, while those with higher humidity required more heat absorption.

A previous study by Munte et al. [61] found that increasing temperatures affect compounds in sago fiber specimens, with the peak of compound changes stopping at approximately 400 °C. This research differs from the previous study in that compound changes continued until temperatures above 600 °C were reached, indicating that the different parts of the sago specimen have unique compound increases and decreases. Furthermore, it demonstrated that adding sago fiber as a composite additive enhances heat resistance at higher temperatures than composites without sago fiber. This study corroborates these findings but focuses on different parts of the sago tree. Another study by Chiang et al. [62] compared the weight of sago in TGA testing and found that a weight of 80 % exhibited superior heat resistance compared to specimens with a weight of 90 %. This research compares the varying mass of different sago tree parts and their impact on thermal resistance.

The samples underwent a drying process, resulting in the decomposition of low-volatile-temperature compounds. This decomposition was observed as a single endothermic peak in the initial stage for all samples tested. The temperature peaks in this zone ranged from 184 to 195 °C for sago bark, 178–193 °C for the outer part of sago, and 176–186 °C for the interior section of sago. Stage II observed most exothermic phenomena, with two distinct peaks. The first peak corresponds to hemicellulose degradation, while the second, higher peak indicates cellulose degradation. These peaks are crucial in biomass degradation, as decomposition during this phase is closely linked to the primary pseudo-components: hemicellulose, cellulose, and lignin [63,64]. These findings align with previous studies that have shown that cellulose decomposes between 230 °C and 450 °C, hemicellulose between 180 °C and 340 °C, and lignin above 500 °C [55,58,65].

In stage III, the DTA profile showed the inner part of the sago had significantly higher fixed carbon content than the outer part and bark. Sago's outer and bark parts, being highly reactive in stage II, contained more volatile materials than the inner part. Higher volatile matter in biomass, due to its reactivity and ease of devolatilization, is preferred for pyrolysis [58]. As ash and fixed carbon increase during pyrolysis, the volatile component of the biochar produced decreases. With more fixed carbon in the biomass material, biochar production increases. Other studies have also observed a rise in fixed carbon content [58,66]. With its higher fixed carbon content, the inner part of sago is favorable for producing activated char, while the bark and outer part are ideal for gasification and pyrolysis applications.

3.3 Comparison based on Proximate and Ultimate Analyses.

**Fig. 8:**
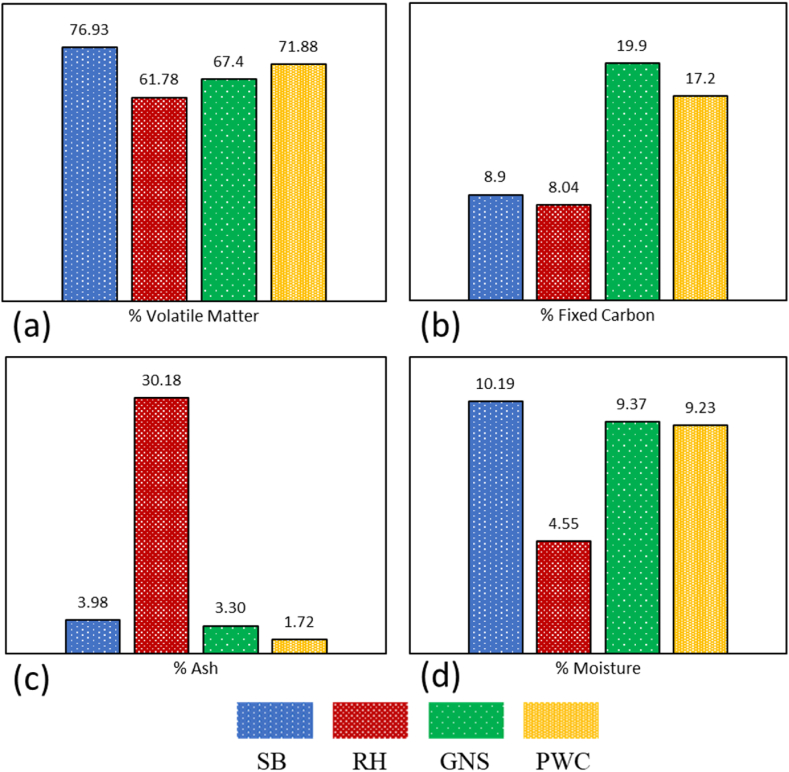
Comparison of (a) volatile matter, (b) fixed carbon, (c) ash, and (d) moisture between different samples: sago bark (SB), rice husk (RH), groundnut shell (GNS), and pinewood chips (PWC).

The proximate and ultimate analysis, as presented in Table 3, compares sago bark with rice husk. This comparison draws upon research from George et al. [68], which used groundnut shell (GNS) and pinewood chips (PWC) as primary materials. The volatile matter test data reveals sago bark's higher value of 76.93 % compared to rice husk's 61.78 %. Meanwhile, GNS and PWC exhibited volatile matter contents of 67.40 wt% and 71.88 wt%, respectively (Fig. 8). These differences in volatile matter contents may be influenced by the moisture content in the specimens. Sago husk contains more fixed carbon (8.90 %) than rice husk (8.04 %). The fixed carbon content affects the heat output produced from the biomass. PWC and GNS exhibited fixed carbon percentages of 17.18 wt% and 19.94 wt%, respectively. This suggests that GNS may produce more char during gasification. The moisture content of sago bark is 10.19 %, higher than rice husk (4.55 %), GNS (9.37 %), and PWC (9.23 %). High moisture content in biomass reduces the yield during combustion. Sago husk's high moisture content necessitates more effort to reduce it. Rice husk has a much higher ash content (30.18 %) compared to sago husk (3.98 %), GNS (3.30 %), and PWC (1.72 %), indicating that rice husk produces more ash, which is mainly a waste by-product of combustion. Detailed results from the testing can be seen in Fig. 8(a–d).

**Table 3:** Proximate and ultimate analysis for sago bark and rice husk [67].

Parameter	Rice Husk (Value)	Sago Bark (Value)	Δ (SB − gns )
***PROXIMATE ANALYSIS*** (%, **ADB**)			
**VOLATILE MATTER**	61.78	76.93	15.15
**FIXED CARBON**	8.04	8.90	0.86
**MOISTURE**	4.55	10.19	5.64
**ASH**	30.18	3.98	−26.2
***ULTIMATE ANALYSIS*** (%, **ADB**)			
**CARBON (C)**	37.65	38.04	0.39
**HYDROGEN (H)**	5.13	5.57	0.44
**NITROGEN (N)**	1.63	0.10	−1.53
**OXYGEN (O)**^a^	55.40	42.09	−13.31
**SULFUR (S)**	0.18	4.73	4.55
***HEATING VALUE*** (mJ/kg)			
***LHV***	12.85	15.12	2.27

a calculated by difference adb (air−dried basis).

**Fig. 9:**
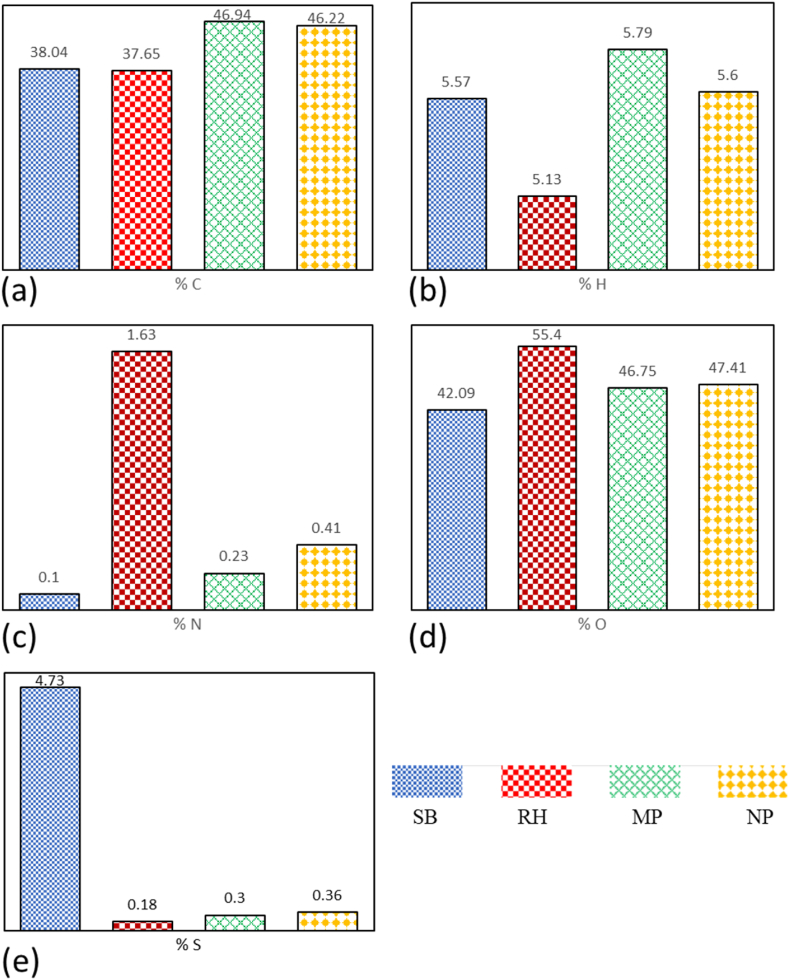
Comparison of (a) carbon, (b) hydrogen, (c) nitrogen, (d) oxygen, and (e) sulfur between sago bark (SB), rice husk RH), *Mbwazirume* peel (MP), and *Nakyinyika* peel (NP).

The ultimate analysis test in Table 3 compares the compound content of rice husk and sago bark. The comparison in Fig. 9(a–e) also considers a study by Yusuf and Inambao [69], where simultaneous combustion was used to degrade biomass fuels made from *Mbwazirume* peel (MP) and *Nakyinyika* peel (NP) for ultimate analysis.

The carbon content test reveals that sago bark has a higher carbon content (38.04 %) compared to rice husk (37.65 %) but lower than MP (46.94 %) and NP (46.22 %). This carbon content influences the energy produced during biomass combustion. In terms of hydrogen content, sago bark ranks third at 5.57 %, following MP (5.79 %) and NP (5.6 %), while rice husk contains 5.13 %. The rice husk has the highest oxygen content at 55.40 %, whereas sago bark has the lowest at 42.09 %. MP and NP have oxygen contents of 46.75 % and 47.41 %. The decline in H and O content in biomass fuels could be attributed to the splitting of weak bonds within the char structure. Regarding nitrogen content, rice husk leads with 1.63 %, while sago bark has the least at 0.10 %. MP and NP have nitrogen contents of 0.23 % and 0.41 %, respectively. Sago bark shows the highest sulfur content at 4.73 %, indicating a potential drawback for biofuel production, as lower sulfur percentages are preferable. In contrast, MP and NP, with sulfur contents of 0.3 % and 0.36 %, respectively, emerge as more reliable candidates for biofuel.

## 4 Conclusions

The characterization and evaluation of sago biomass from the Bata, Yakari, Dondo, and Yebha districts in Sentani, Papua, have been successfully completed. SEM–EDX analysis revealed that O, C, and Si were the predominant elements, with carbon content ranging from 30.75 % to 38.87 %. This range suggests that all parts of the sago plant hold potential as biomass fuel. DTA and TGA tests successfully predicted the cellulose, hemicellulose, and lignin content in different parts of the sago tree (inner, outer, and bark). The bark section exhibited the highest moisture content at 55 %, followed by the outer part at 42.6 % and the inner part at 13.3 %. The inner part contained the highest lignin content compared to the bark and outer part of the sago. The outer and bark parts of the sago were more reactive in stage II of the DTA profile, indicating higher cellulose and hemicellulose content. These components, being dominant compared to lignin, are suitable for gasification and pyrolysis. The inner part of the sago, with its higher fixed carbon content, is desirable for producing activated char. These results strengthen the waste-to-energy process, which is of significant interest and further highlights the potential of using renewable energy sources. Additionally, this research provides valuable insights for future studies on gasification and biomass characterization processes applicable in gasification, co-firing, or alternative energy and electricity generation.

## Data availability statement

The data will be made available on request.

## CRediT authorship contribution statement

**Benny Susanto:** Writing - review & editing, Writing - original draft, Visualization, Validation, Software, Resources, Project administration, Methodology, Investigation, Funding acquisition, Formal analysis, Data curation, Conceptualization. **Yohanis Tangke Tosuli:** Writing - review & editing. **Adnan:** Writing - review & editing, Writing - review & editing. **Cahyadi:** Writing - review & editing. **Hossein Nami:** Writing - review & editing. **Adi Surjosatyo:** Writing - review & editing, Supervision. **Daffa Alandro:** Writing - review & editing. **Alvin Dio Nugroho:** Writing - review & editing. **Muhammad Ibnu Rashyid:** Writing - review & editing. **Muhammad Akhsin Muflikhun:** Writing - review & editing, Writing - original draft, Supervision.

## Declaration of competing interest

The authors declare that they have no known competing financial interests or personal relationships that could have appeared to influence the work reported in this paper.

## Figures and Tables

**Fig. A 1:**
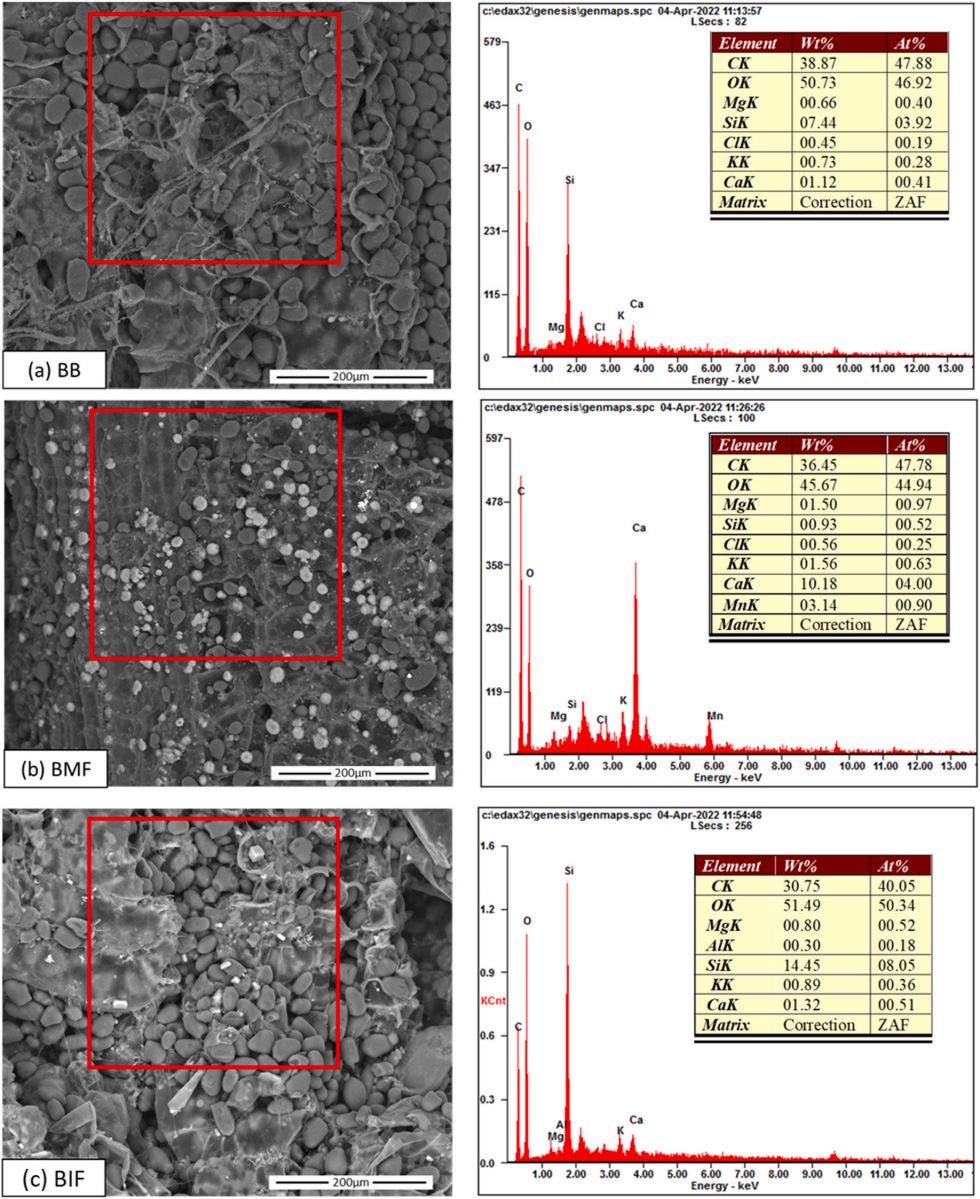

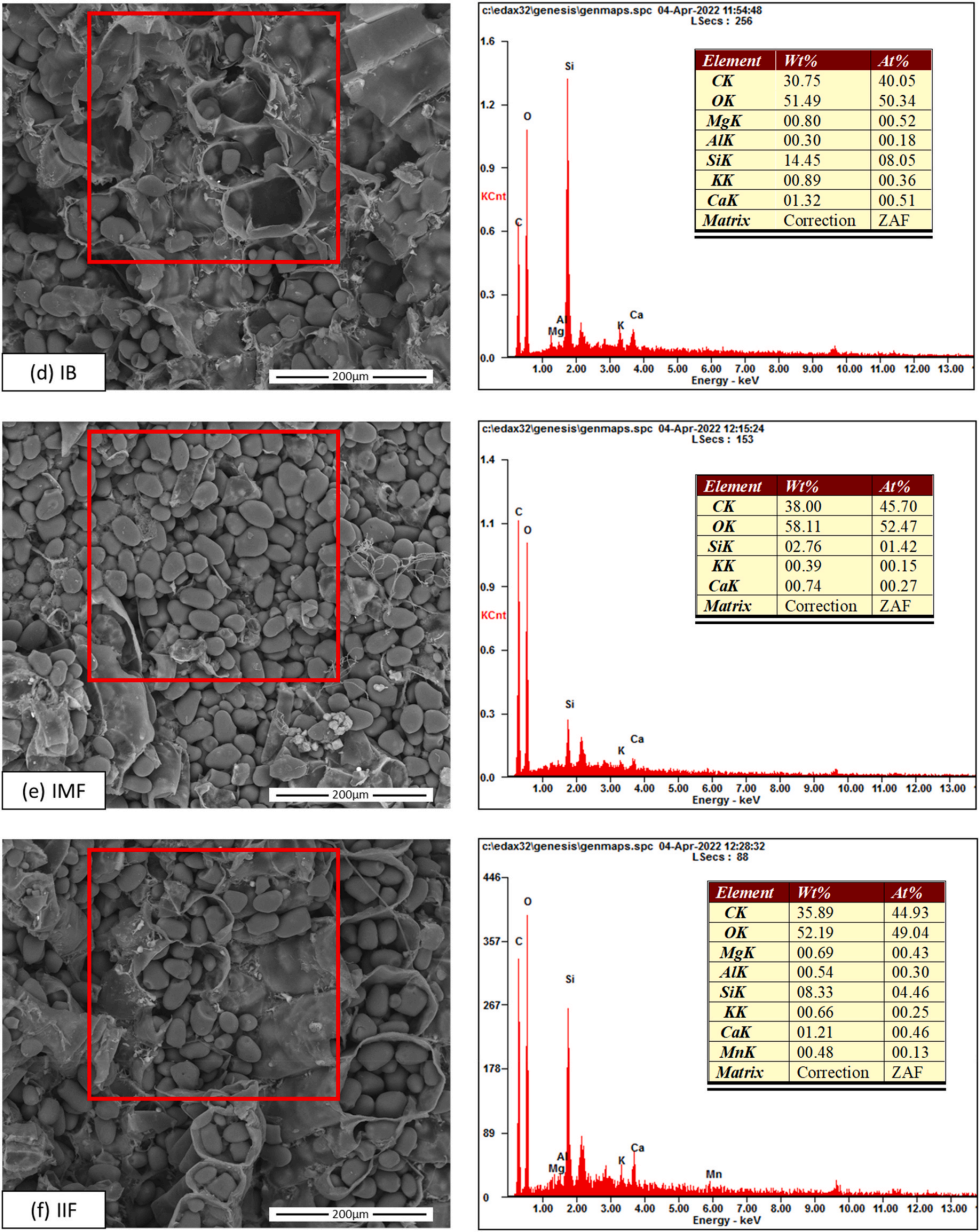

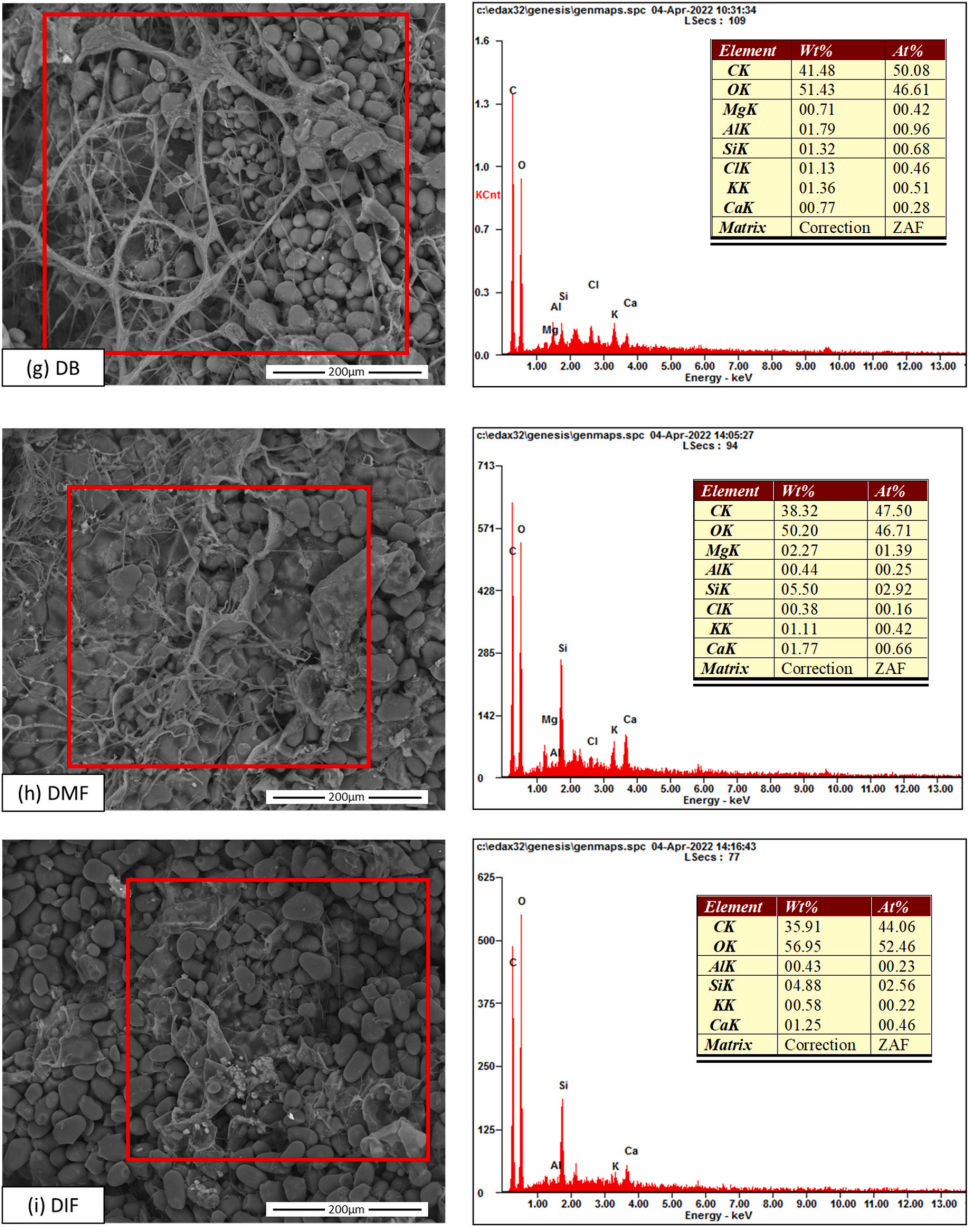

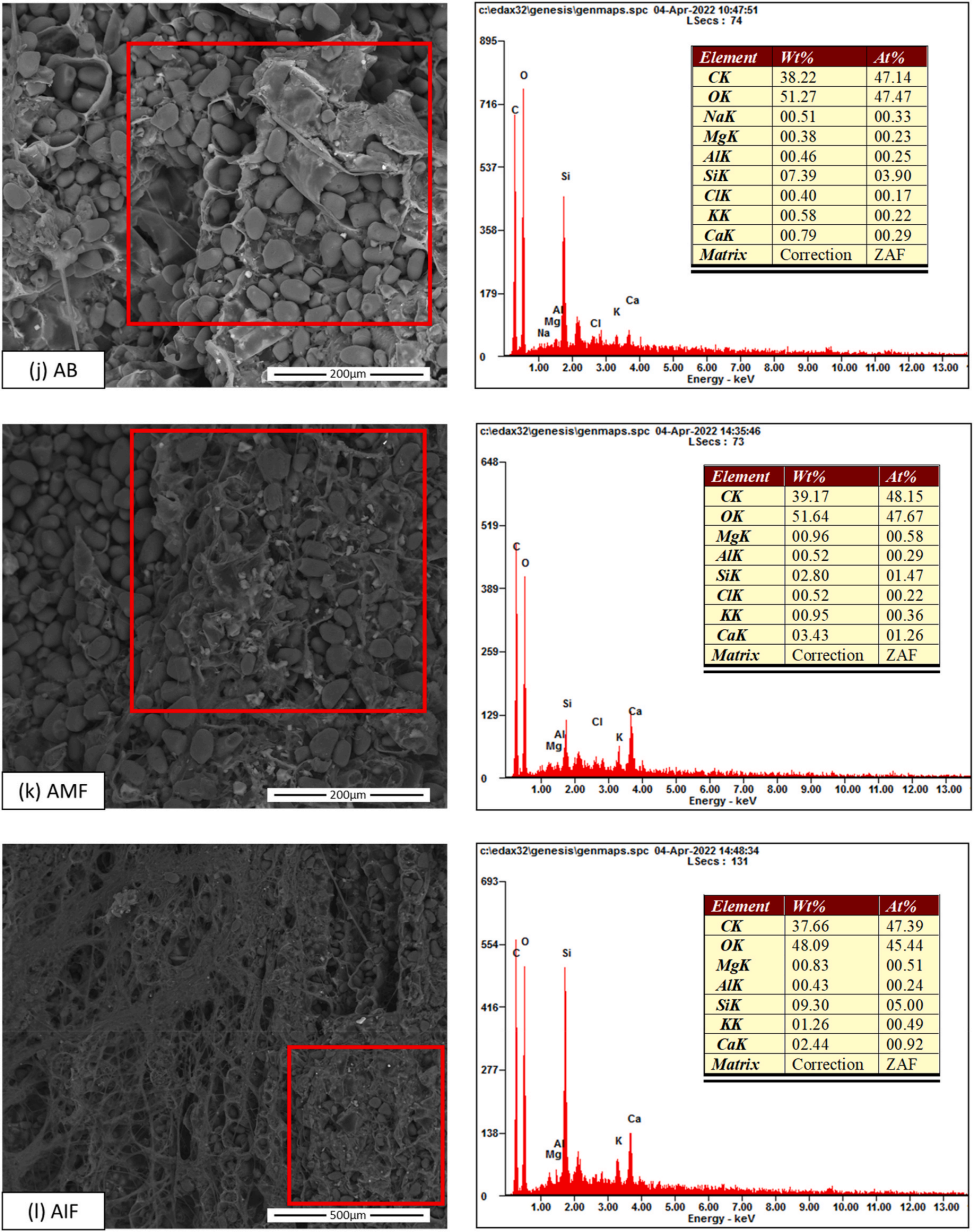
SEM-EDX location and chemical composition from representative samples of (a) BB, (b) BMF, (c) BIF, (d)IB, (e) IMF, (f) IIF, (g) DB, (h) DMF, (i) DIF, (j) AB, (k) AMF, (l) AIF.

## Appendix

SEM-EDX results from all combinations of samples.

## References

[1] Greenhill A.R., Shipton W.A., Blaney B.J., Warner J.M. (2007). Fungal colonization of sago starch in Papua New Guinea. Int. J. Food Microbiol..

[2] Kuroda K.I., Ozawa T., Ueno T. (2001). Characterization of sago palm (Metroxylon sagu) lignin by analytical pyrolysis. J. Agric. Food Chem..

[3] Syauqiah I., Sir Kautsar Harivram A., Lulu Atika Rampun E., Amryna Chairul Putri D., Safitri N.G., Elma M. (2022). Removal of artificial iron ions using activated carbon from sago pith waste. Mater. Today Proc..

[4] Nguyen P.X.T., Ho K.H., Do N.H.N., Nguyen C.T.X., Nguyen H.M., Tran K.A., Le K.A., Le P.K. (2022). A comparative study on modification of aerogel-based biosorbents from coconut fibers for treatment of dye- and oil-contaminated water. Materials Today Sustainability.

[5] Letsoin S.M.A., Purwestri R.C., Rahmawan F., Herak D. (2022). Recognition of sago palm trees based on transfer learning. Rem. Sens..

[6] Letsoin S.M.A., Herak D., Rahmawan F., Purwestri R.C. (2020). Land cover changes from 1990 to 2019 in Papua, Indonesia: results of the remote sensing imagery. Sustainability.

[7] Sidiq F.F., Coles D., Hubbard C., Clark B., Frewer L.J. (2021). Sago and the indigenous peoples of Papua, Indonesia: a review. Journal of Agriculture and Applied Biology.

[8] Yukiootoyoda H., Johnson D. (2018).

[9] Trisia M.A., Takeshita H., Kikuta M., Ehara H. (2020). Factors determining sago starch import demand: empirical evidence from Japan. Journal of International Logistics and Trade.

[10] Dewayani W., Suryani, Arum R.H., Septianti E. (2022). IOP Conf Ser Earth Environ Sci.

[11] Trisia M.A., Tachikawa M., Ehara H. (2021). The role of the sago supply chain for rural development in Indonesia: a review and perspective. Reviews in Agricultural Science.

[12] Ambariyanto P. Hariyadi (2017).

[13] Abbas B., Dailami M., Santoso B. (2017). Munarti, genetic variation of sago palm (metroxylonsagu rottb.) progenies with natural pollination by using RAPD markers. Nat. Sci..

[14] Karim A.A., Tie P.-L., Manan D.M.A., Zaidul I.S.M. (2008). Starch from the sago (metroxylon sagu) palm tree-properties, prospects, and challenges as a new industrial source for food and other uses. Compr. Rev. Food Sci. Food Saf..

[15] Bocobo Aljon E., Maureal Juliet R., Sajonia Anamarie P. (2022). Proceedings of International Exchange and Innovation Conference on Engineering & Sciences (IEICES).

[16] Awg-Adeni D.S., Abd-Aziz S., Bujang K., Hassan M.A. (2016). Bioconversion of sago residue into value added products. Afr. J. Biotechnol..

[17] Wibowo A., Alandro D., Killian M.S., Nugroho G., Raghu S.N.V., Akhsin Muflikhun M. (2023). Mechanical evaluation and characterization of hybrid sugarcane bagasse microfibrillated cellulose with added filler materials for use as disposable utensils. Adv. Compos. Mater..

[18] Hao M., Hu Y., Wang B., Liu S. (2017). Mechanical behavior of natural fiber-based isogrid lattice cylinder. Compos. Struct..

[19] Lotfi A., Li H., Dao D.V., Prusty G. (2019). Natural fiber–reinforced composites: a review on material, manufacturing, and machinability. J. Thermoplast. Compos. Mater..

[20] Sumesh K.R., Kanthavel K., Kavimani V. (2020). Peanut oil cake-derived cellulose fiber: extraction, application of mechanical and thermal properties in pineapple/flax natural fiber composites. Int. J. Biol. Macromol..

[21] Li M., Pu Y., Thomas V.M., Yoo C.G., Ozcan S., Deng Y., Nelson K., Ragauskas A.J. (2020). Recent advancements of plant-based natural fiber–reinforced composites and their applications. Compos. B Eng..

[22] Das S.C., Paul D., Grammatikos S.A., Siddiquee M.A.B., Papatzani S., Koralli P., Islam J.M.M., Khan M.A., Shauddin S.M., Khan R.A., Vidakis N., Petousis M. (2021). Effect of stacking sequence on the performance of hybrid natural/synthetic fiber reinforced polymer composite laminates. Compos. Struct..

[23] Zhao X., Copenhaver K., Wang L., Korey M., Gardner D.J., Li K., Lamm M.E., Kishore V., Bhagia S., Tajvidi M., Tekinalp H., Oyedeji O., Wasti S., Webb E., Ragauskas A.J., Zhu H., Peter W.H., Ozcan S. (2022). Recycling of natural fiber composites: challenges and opportunities. Resour. Conserv. Recycl..

[24] Prabhu L., Krishnaraj V., Gokulkumar S., Sathish S., Ramesh M. (2019). Mechanical, chemical and acoustical behavior of sisal - tea waste - glass fiber reinforced epoxy based hybrid polymer composites. Mater. Today Proc..

[25] Ravi Y.V., Kapilan N., Rajole S., Balaji Y.S., Varun Kumar Reddy N., Venkatesha B.K. (2021). Damage resistance evaluation of E-glass and hybrid hemp-banana natural fiber composite helmet using drop weight impact test. Mater. Today Proc..

[26] Singh T. (2021). Optimum design based on fabricated natural fiber reinforced automotive brake friction composites using hybrid CRITIC-MEW approach. J. Mater. Res. Technol..

[27] shariff M., Nagamadhu M., Jaiprakash M., Karthikeyan K., Kiran (2020). Effect of drilling process parameters on natural fiber reinforced basket epoxy composites using grey relational analysis. Mater. Today Proc..

[28] Prabhu L., Krishnaraj V., Sathish S., Gokulkumar S., Karthi N., Rajeshkumar L., Balaji D., Vigneshkumar N., Elango K.S. (2021). A review on natural fiber reinforced hybrid composites: chemical treatments, manufacturing methods and potential applications. Mater. Today Proc..

[29] Ahmad J., Zhou Z. (2022). Mechanical properties of natural as well as synthetic fiber reinforced concrete: a review. Constr Build Mater.

[30] Nuryanta M.I., Sentanuhady J., Muflikhun M.A. (2022). Moisture absorption behavior of hybrid composite laminates consist of natural and glass fiber. Mater. Today Proc..

[31] Nugraha A.D., Nuryanta M.I., Sean L., Budiman K., Kusni M., Muflikhun M.A. (2022). Recent progress on natural fibers mixed with CFRP and GFRP: properties, characteristics, and failure behaviour. Polymers.

[32] Nuryanta M.I., Aryaswara L.G., Korsmik R., Klimova-Korsmik O., Nugraha A.D., Darmanto S., Kusni M., Muflikhun M.A. (2023). The interconnection of carbon active addition on mechanical properties of hybrid agel/glass fiber-reinforced green composite. Polymers.

[33] Jain D., Kamboj I., Bera T.K., Kang A.S., Singla R.K. (2019). Experimental and numerical investigations on the effect of alkaline hornification on the hydrothermal ageing of Agave natural fiber composites. Int J Heat Mass Transf.

[34] Kumagai A., Tajima N., Iwamoto S., Morimoto T., Nagatani A., Okazaki T., Endo T. (2019). Properties of natural rubber reinforced with cellulose nanofibers based on fiber diameter distribution as estimated by differential centrifugal sedimentation. Int. J. Biol. Macromol..

[35] Manimaran P., Senthamaraikannan P., Sanjay M.R., Marichelvam M.K., Jawaid M. (2018). Study on characterization of Furcraea foetida new natural fiber as composite reinforcement for lightweight applications. Carbohydr. Polym..

[36] Araya-Letelier G., Concha-Riedel J., Antico F.C., Valdés C., Cáceres G. (2018). Influence of natural fiber dosage and length on adobe mixes damage-mechanical behavior. Constr Build Mater.

[37] Khan T., Sultan M.T.B.H., Ariffin A.H. (2018). The challenges of natural fiber in manufacturing, material selection, and technology application: a review. J. Reinforc. Plast. Compos..

[38] Tenriawaru E.P., Supu I., Cambaba S. (2018). IOP Conf Ser Earth Environ Sci.

[39] Rasyid T.H., Kusumawaty Y., Hadi S. (2020). IOP Conf Ser Earth Environ Sci.

[40] Siruru H., Syafii W., Wistara I.N.J., Pari G. (2019). Characteristics of metroxylon rumphii (pith and bark waste) from seram island, Maluku, Indonesia. Biodiversitas.

[41] Wahi R., Chuah Abdullah L., Nourouzi Mobarekeh M., Ngaini Z., Choong Shean Yaw T. (2017). Utilization of esterified sago bark fibre waste for removal of oil from palm oil mill effluent. J. Environ. Chem. Eng..

[42] Du C., Jiang F., Jiang W., Ge W., kui Du S. (2020). Physicochemical and structural properties of sago starch. Int. J. Biol. Macromol..

[43] Du C., Jiang F., Jiang W., Ge W., kui Du S. (2020). Physicochemical and structural properties of sago starch. Int. J. Biol. Macromol..

[44] Ahmad A.N., Lim S.A., Navaranjan N., Hsu Y.I., Uyama H. (2020). Green sago starch nanoparticles as reinforcing material for green composites. Polymer (Guildf).

[45] (2012). SUMARYONO, W. MUSLIHATIN, D.I.A.H. RATNADEWI, effect of carbohydrate source on growth and performance of in vitro sago palm (metroxylon sagu rottb.) plantlets. Hayati.

[46] Zhu F. (2019). Recent advances in modifications and applications of sago starch. Food Hydrocoll.

[47] Wardono H.P., Agus A., Astuti A., Ngadiyono N., Suhartanto B. (2021). E3S Web of Conferences.

[48] Yamamoto Y. (2019). Evaluation and development of sago palm as a natural starch resource in the tropics. Sago Palm.

[49] Trisia M.A., Ehara H. (2021). Commercialization of sago starch in Indonesia: production, consumption and international trading. Sago Palm.

[50] Yamamoto Y., Katayama K., Yoshida T., Miyazaki A., Jong F.S., Pasolon Y.B., Matanubun H., Rembon F.S., Nicholus, Limbongan J. (2020). Changes in leaf and trunk characteristics related to starch yield with age in two sago palm folk varieties grown near Jayapura, Papua, Indonesia. Tropical Agriculture and Development.

[51] Tibayan E.B., Muﬂikhun M.A., Villagracia A.R.C., Kumar V., Santos G.N.C. (2020). Structures and UV resistance of Ag/SnO2 nanocomposite materials synthesized by horizontal vapor phase growth for coating applications. J. Mater. Res. Technol..

[52] Foronda J.R.F., Aryaswara L.G., Santos G.N.C., Raghu S.N.V., Muflikhun M.A. (2023). Broad-class volatile organic compounds (VOCs) detection via polyaniline/zinc oxide (PANI/ZnO) composite materials as gas sensor application. Heliyon.

[53] Singhal R.S., Kennedy J.F., Gopalakrishnan S.M., Kaczmarek A., Knill C.J., Akmar P.F. (2008). Industrial production, processing, and utilization of sago palm-derived products. Carbohydr. Polym..

[54] Muflikhun M.A., Yokozeki T. (2023). Systematic analysis of fractured specimens of composite laminates: different perspectives between tensile, flexural, Mode I, and Mode II test. International Journal of Lightweight Materials and Manufacture.

[55] El-Sayed S.A., Mostafa M.E. (2015). Kinetic parameters determination of biomass pyrolysis fuels using TGA and DTA techniques. Waste Biomass Valorization.

[56] Yu S., Yang X., Li Q., Zhang Y., Zhou H. (2023). Breaking the temperature limit of hydrothermal carbonization of lignocellulosic biomass by decoupling temperature and pressure. Green Energy Environ..

[57] Yu S., Dong X., Zhao P., Luo Z., Sun Z., Yang X., Li Q., Wang L., Zhang Y., Zhou H. (2022). Decoupled temperature and pressure hydrothermal synthesis of carbon sub-micron spheres from cellulose. Nat. Commun..

[58] Kumar P., Subbarao P.M.V., Kala L.D., Vijay V.K. (2021). Thermogravimetry and associated characteristics of pearl millet cob and eucalyptus biomass using differential thermal gravimetric analysis for thermochemical gasification. Therm. Sci. Eng. Prog..

[59] Chiang T.C., Hamdan S., Osman M.S. (2016). Urea formaldehyde composites reinforced with sago fibres analysis by FTIR, TGA, and DSC. Adv. Mater. Sci. Eng..

[60] Munte S., Banjarnahor M., Tanjung D.A., Budi R.S. (2023). MECHANICAL test and thermal stability on thermoplastic sago (metroxylon sagu rottb.) combination of polyethylene and polypropylene. Rasayan Journal of Chemistry.

[61] Munte S., Banjarnahor M., Tanjung D.A., Budi R.S. (2023). MECHANICAL test and thermal stability on thermoplastic sago (metroxylon sagu rottb.) combination of polyethylene and polypropylene. Rasayan Journal of Chemistry.

[62] Chiang T.C., Hamdan S., Osman M.S. (2016). Urea formaldehyde composites reinforced with sago fibres analysis by FTIR, TGA, and DSC. Adv. Mater. Sci. Eng..

[63] Baeza Jaime (2001).

[64] Claoston N., Samsuri A.W., Ahmad Husni M.H., Mohd Amran M.S. (2014). Effects of pyrolysis temperature on the physicochemical properties of empty fruit bunch and rice husk biochars. Waste Manag. Res..

[65] Lyon R.E. (1997).

[66] Parascanu M.M., Sandoval-Salas F., Soreanu G., Valverde J.L., Sanchez-Silva L. (2017). Valorization of Mexican biomasses through pyrolysis, combustion and gasification processes. Renew. Sustain. Energy Rev..

[67] Ma Z., Ye J., Zhao C., Zhang Q. (2015). Gasification of rice husk in a downdraft gasifier: the effect of equivalence ratio on the gasification performance, properties, and utilization analysis of. Byproducts of Char and Tar.

[68] George O.S., Dennison M.S., Yusuf A.A. (2023). Characterization and energy recovery from biomass wastes. Sustain. Energy Technol. Assessments.

[69] Yusuf A.A., Inambao F.L., Hassan A.S., Nura S.S., Karthickeyan V. (2020). Comparative study on pyrolysis and combustion behavior of untreated Matooke biomass wastes in East Africa via TGA, SEM, and EDXS. International Journal of Energy and Environmental Engineering.

